# Exploration of Exosomal miRNAs from Serum and Synovial Fluid in Arthritis Patients

**DOI:** 10.3390/diagnostics12020239

**Published:** 2022-01-19

**Authors:** Yingying Xie, Wenwen Chen, Mengqian Zhao, Yuhai Xu, Hao Yu, Jianhua Qin, Hongjing Li

**Affiliations:** 1 Dalian Institute of Chemical Physics, Chinese Academy of Sciences, Dalian 116023, China; xieyy@dicp.ac.cn (Y.X.); chenwenwen@dicp.ac.cn (W.C.); mengqianzhao@dicp.ac.cn (M.Z.); yuhao@dicp.ac.cn (H.Y.); 2 University of Chinese Academy of Sciences, Beijing 100864, China; 3 First Affiliated Hospital of Dalian Medical University, Dalian Medical University, Dalian 116011, China; lzn04@163.com

**Keywords:** exosomes, miRNA, synovial fluid, arthritis

## Abstract

Arthritis is caused by inflammation, infection, degeneration, trauma, or other factors that affect approximately 250 million people all over the world. Early diagnosis and prediction are essential for treatment. Exosomes are nanoscale vesicles that participate in the process of joint disease. Serum is the mainly used sources in the study of arthritis-related exosomes, while whether serum exosomes can reflect the contents of synovial fluid exosomes is still unknown. In this work, we separated exosomes from serum and the synovial fluid of osteoarthritis patients and compared their miRNA expression utilizing miRNA sequencing. The results revealed that 31 upregulated and 33 downregulated miRNAs were found in synovial fluid compared to serum. Transcriptome analysis showed that these differentially expressed miRNAs were mainly associated with intercellular processes and metabolic pathways. Our results show that serum-derived exosomes cannot fully represent the exosomes of synovial fluid, which may be helpful for the study of joint diseases and the discovery of early diagnostic biomarkers of arthritis.

## 1. Introduction

Arthritis is mainly related to autoimmune reaction, infection, metabolic disorder, trauma, degenerative lesions, and other factors [1]. With the combined impact of global population aging and increasing obesity, this already cumbersome syndrome is becoming increasingly common, affecting 250 million people worldwide [2]. Drugs for osteoarthritis patients can only relieve pain without actually preventing progression of the disease [3]. Early diagnosis and prediction are very important for treatment. Exosomes are one kind of extracellular vesicle (EV) with a size range from 40 to 160 nm in diameter [4]. All kinds of cells can release exosomes, and they are found in almost all kinds of body fluids such as blood [5,6,7], urine [8,9], sweat [10], saliva [11], milk [12], and synovial fluid. They play an important role in information transmission, metabolism, disease processes, and immune regulation in the human body. In addition, they have been used as important biomarkers in liquid biopsies for the early clinical diagnosis of cancer, evaluation of drug efficacy, and monitoring of disease progress [13,14].

In recent years, exosomes have been used in research on arthritis-related diseases, especially in the study of exosomal miRNAs. miRNAs are a class of highly conserved and endogenous non-coding small RNAs with a length of 18–24 nucleotides [15], and they have been used as potential biomarkers and are the most widely studied molecules in exosomes [16]. As the most commonly used body fluid for testing, serum has been reported to test exosomal miRNAs in patients with arthritis [17,18]. At the same time, synovial fluid—the fluid in the joint cavity—has also been reported in exosomal miRNA analysis [19]. However, the difference and correlation between miRNAs in serum exosomes and synovial fluid exosomes and whether serum exosomes can reflect the situation in the joint cavity are still unknown. In this work, we separated exosomes from serum and the synovial fluid of osteoarthritis patients and explored their miRNAs’ expression by miRNA sequencing.

## 2. Materials and Methods

### 2.1. Fabrication of the Exosome Separation Device

The exosome separation device was composed of four parts: chitosan scaffolds, reaction tubes, working buffer, and a shaker (NuoMi, Taizhou, China). The chitosan scaffold was used to provide the chitosan substrates with large specific surface area. The working buffer was used to create an acid reaction environment and wash away unabsorbed impurities. The reaction tubes were used to combine samples with chitosan scaffolds, and the shaker was used to enhance the reaction between the samples and chitosan scaffolds.

The working buffer was 10 mM MES (2-(N-morpholino) ethanesulfonic acid, Aladdin, Shanghai, China) with sodium hydroxide to adjust the pH to 6.0. The chitosan scaffolds were synthesized by the classic freeze-drying method. Two percent chitosan (Aladdin, 100–200 mPa·s, Shanghai, China) was first mixed with 5% DMSO (dimethyl sulfoxide) in 1% acetic acid. Then, equal volumes of 0.3% glutaraldehyde were added to the solution for crosslinking chitosan. After the reaction was completed at −20 °C for over 16 h, 1% NaBH_4_ solution was used to remove the unreacted reagent three times followed by washing with deionized water three times. Finally, the products were put into a lyophilizer to obtain dried chitosan scaffolds.

### 2.2. Sources and Storage Conditions of Human Serum and Synovial Fluid

Clinical serum samples and synovial fluid samples of osteoarthritis patients were obtained from the First Affiliated Hospital of Dalian Medical University based on the protocols authorized by the institutional review committee of the First Affiliated Hospital of Dalian Medical University (PJ-KY-2019-96(X), 29 October 2019). After collection from the donors, all samples were transferred to a −80 °C refrigerator as soon as possible until the experiment started.

### 2.3. Exosome Collection from Cell Culture Medium

The medium used in this experiment was from C2C12 (mouse muscle cell line). The culture medium for this cell line is high glucose DMEM combined with 1% penicillin–streptomycin and 10% FBS (*v*/*v*). When the bottom of the Petri dish was full of cells, the medium was collected for exosome separation. Cell culture-related reagents were all purchased from Gibco, New York, NY, USA.

For the preparation of pretreated medium, the obtained medium was centrifuged at 1000× *g* for 10 min and then 10,000× *g* for 30 min at 4 °C to remove cells, cell fragments, and large vesicles. Then, a commercial 220 nm PVDF filter membrane (Millex, Atlanta, GA, USA) was used to filter the supernatant to remove substances larger than 220 nm. For the preparation of standard samples through ultracentrifugation, the filtrate was ultracentrifuged to precipitate exosomes at the speed of 120,000× *g* for 75 min at 4 °C. Then, using working buffer to resuspend exosomes followed by ultracentrifugation at the speed of 120,000× *g* for 75 min at 4 °C to precipitate exosomes. The precipitate was resuspended with 200 μL MES and stored in refrigerator with the temperature of −80 °C.

### 2.4. Pretreatment of Clinical Samples

The pretreatment method for the clinical samples was approximately the same as for the culture medium. The thawed serum and synovial fluid were centrifuged at 4 °C, 1000× *g* for 10 min followed by 10,000× *g* for 30 min. Then, in order to reduce the loss of filtration due to the rarity of clinical samples, working buffer was used to dilute and adjust the pH of samples at a volume ratio of 1:4 (sample: working buffer). The mixture was subsequently filtered through a 220 nm filter.

### 2.5. Isolation of Exosomes Using the Exosome Separation Device

Samples and chitosan scaffolds were added to the reaction tube and then mixed on a shaker at 4 °C for 20 min to capture exosomes via electrostatic adsorption. Then, using working buffer, unreacted exosomes and impurities were washed away three times. The exosomes were absorbed on the chitosan scaffolds. The shaker had a fixed swing angle of 15 °C and was ran at a speed of 30 rpm/min.

### 2.6. Quantitative Analysis of Exosomes

A fluorometer (Qubit 3.0, Waltham, MA, USA) was used to quantify the concentration of exosomes. The capture efficiency was the ratio of the concentration difference before and after capture to the initial concentration.

### 2.7. Particle Size Analysis

A nano-laser particle detector (Zetasizer Nano, Malvern, UK) was used to characterize the particle size distribution of exosomes. The temperature was 25 °C. The material RI was 1.59. The duration used was 500 s, and the measurement position was 4.65.

### 2.8. Transmission Electron Microscopy Image

Exosomes were purified from cell culture medium by ultracentrifugation, as described above, and resuspended in PBS. Before staining, exosomes were fixed in 4% paraformaldehyde for 30 min at 4 °C. Afterwards, exosomes were loaded on a copper grid for 5 min, and the excess liquid was removed with filter paper. Finally, the exosomes were stained with uranyl acetate (Damao, Tianjin, China) for 2 min before examination under a transmission electron microscope.

### 2.9. Scanning Electron Microscopy Image

For morphology analysis, chitosan scaffolds with/without exosomes were fixed with 4% paraformaldehyde (Damao, Tianjin, China) and dehydrated in a series of alcohol solutions (25%, 50%, 75%, 90%, 95%, and 100% alcohol in PBS). Then, the samples were dried naturally at ambient temperature and, subsequently, coated with Pt using a coating instrument (Leica EM ACE200, Wetzlar, Germany) before imaging by scanning electron microscope ((JSM-7800F, Akishima-Shi, Japan).

### 2.10. Western Blot Analysis

To extract proteins, chitosan scaffolds with exosomes were lysed in RIPA lysis buffer which contained 1% RMSF and on 1% protease inhibitor ice. Protein lysates were handled by SDS-PAGE (10% gel, 100 V) and transferred onto a NC membrane (Millipore, Burlington, MA, USA). After blocking for 1 h, the membrane was incubated with antibodies against CD63 and CD9 (Abcam, Cambridge, UK) in PBS at 4 °C overnight. Then, the membrane was first washed with PBS three times to remove unreacted antibodies, and horseradish peroxidase conjugated secondary antibodies were used to incubate the membrane for 1 h. Chemiluminescence could be detected by a multifunctional imager (FUSION FX7, Paris, France). The reagents used in the Western blot were mostly purchased from Beyotime, Shanghai, China, except for the antibodies.

### 2.11. RNA Extraction and Quantification

The RNAs in exosomes were extracted using the Trizol method. In brief, 1 mL of TRIzol (Invitrogen, Carlsbad, CA, USA) was added to the samples to break exosomes and release RNAs on ice. Then, chloroform was used to extract phenol and centrifugated at 12,000× *g* for 15 min. The RNAs were in the upper aqueous phase. The upper liquid was carefully absorbed into a new tube, and an equal volume of isopropyl alcohol was added to resuspend the RNAs. After centrifugation at 12,000× *g* for 10 min, 75% methanol was used to wash the precipitate. Afterwards, the RNAs were precipitated by centrifugation at 12,000× *g* for 5 min. The precipitate was resuspended with 10 uL DEPC water, and the concentration of RNAs was determined by means of a spectrophotometer (Thermo Fisher Scientific Inc., Waltham, MA, USA).

### 2.12. miRNA Sequencing

The obtained exosomal RNA was delivered to a sequencing company (Wuhan SeqHealth Technology Corporation, Wuhan, China) on dry ice, where a transcriptome sequencing project was completed on an Illumina paired-end sequencing platform, and the transcriptome data were analyzed by bioinformatic methods.

### 2.13. Data Analysis

All measurements were performed at least three times, and the data are shown as the mean ± SD. The Student’s *t*-test was used to analyze the statistical significance of the two groups. A *p*-value less than 0.05 was considered statistically significant. For the miRNA analysis, a sequencing company conducted the quality control of the original data, and all analyses between samples were based on the sequencing results.

## 3. Results and Discussion

### 3.1. Design of the Exosome Separation Device

**Figure 1:**
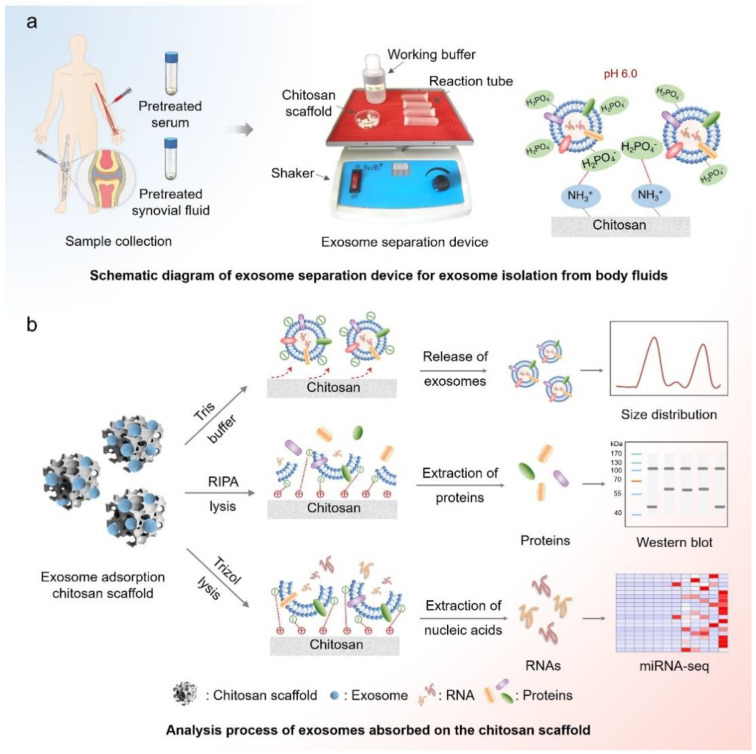
An illustration of isolating exosomes from serum and synovial fluid using the exosome separation device. (**a**) Schematic diagram of the exosome separation device for exosome isolation from the serum and synovial fluid of a human. The device is composed of a chitosan scaffold, reaction tube, working buffer, and a shaker. In an acidic environment, –NH_3_^+^ on chitosan can combine with anionic phosphate groups on a phospholipid bilayer of exosomes thus absorbing exosomes on its surface. (**b**) Analysis process of exosomes absorbed on a chitosan scaffold. The exosomes captured by the chitosan scaffold can be released by alkaline buffer for particle size detection or lysed online for nucleic acids and proteins analysis.

Exosomes are nanoscale microvesicles selected by most types of cells, and they play important roles in various pathological processes. Separating exosomes from biological samples is challenging because of their small size (i.e., 40–160 nm) and similar density with body fluids. In this work, we used a simple strategy that we fabricated and optimized previously [20] to separate exosomes from serum and synovial fluid. This exosome separation device cooperatively integrates scaffold substrates, electrostatic adsorption, and shuttle flow to enable efficient isolation of exosomes from biological samples. As shown in Figure 1a, the main principle of this device is the positive and negative charges’ reactions. In an acidic reaction environment, –NH_2_ groups on chitosan scaffolds will be protonated into –NH_3_^+^ groups; then, the negatively charged phosphate groups of the exosome membrane can be combined onto the surface of the chitosan scaffolds.

The exosomes absorbed onto the chitosan scaffolds can be released by Tris buffer (pH = 8.0) for particle size analysis as we reported in our previous work [21]. This is mainly because the –NH_3_^+^ groups will be deprotonated into –NH_2_ groups in the alkaline solution, and exosomes will be desorbed from the surfaces of chitosan scaffolds. Furthermore, exosomes can also be lysed in situ by RIPA for protein analysis or by TRIzol for RNA analysis (Figure 1b).

### 3.2. Performance of the Exosome Separation Device

**Figure 2:**
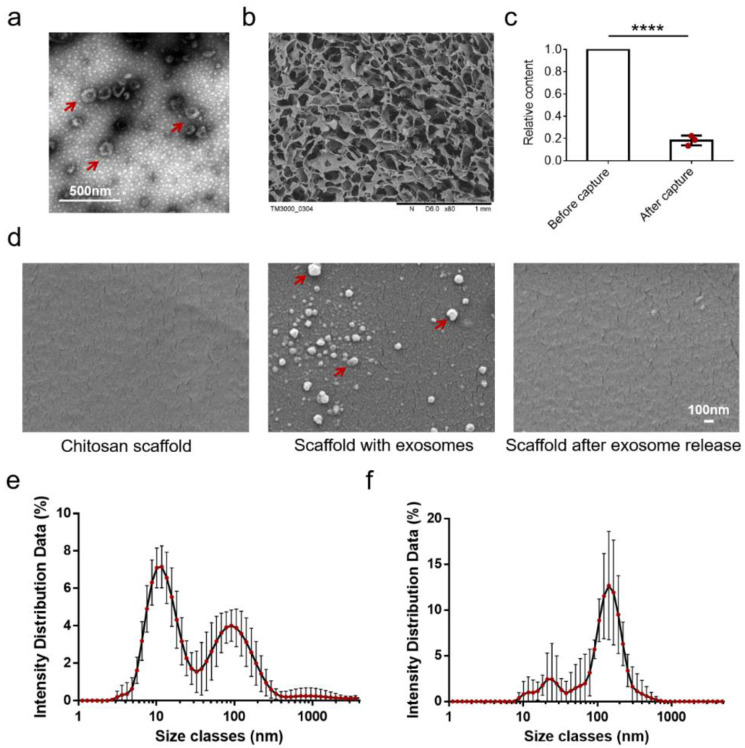
Performance of the exosome separation device. (**a**) TEM images of the structure of standard exosome samples obtained by ultracentrifugation. The red arrows indicate exosomes. (**b**) SEM images of the structure of the chitosan scaffold. (**c**) The relative content of exosomes before and after being separated by the exosome separation device. The results were normalized to the original concentration. The data were presented as the mean ± SD, *n* = 3. **** *p* < 0.0001 by two-sided paired Student’s *t*-test. (**d**) SEM images of the surface of the chitosan scaffold before exosome capture, after exosome capture, and after exosome release. The red arrows indicate exosomes absorbed on the scaffolds. (**e**) Particle size analysis of pretreated cell culture medium samples. The data were presented as mean ± SD, *n* = 3. (**f**) Particle size distribution of exosomes after being isolated by the exosome separation device. The data are presented as the mean ± SD, *n* = 3.

In order to test the performance of the exosome separation device for exosome isolation, we used exosomes acquired from cell culture medium by ultracentrifugation as a standard sample (Figure 2a). The morphology of a chitosan scaffold is shown in Figure 2b. It can be seen that there was an obvious through-hole structure on the chitosan scaffold, which is conducive for liquid exchange.

For the capture efficiency of this device, we used this device to separate exosomes from standard samples and detected the concentration of samples before and after capture. We normalized the concentration of the samples after capture to the original standard samples due to the fact that the concentration of the standard samples in each experiment could not be completely consistent. The result is shown in Figure 2c. The relative content of exosomes in the solution after capture was 0.18 ± 0.04, which means that the capture efficiency of the device was 82 ± 4%. This result is consistent with our previous work, which indicates that the exosome separation device has a stable and reliable performance.

We also used SEM to observe the absorption of exosomes on the surface of chitosan scaffolds. As shown in Figure 2d, the surface of the chitosan scaffold was smooth, while a large number of small particles can be observed on the surface of the chitosan scaffold after exosome adsorptions (medium). The size of the particles were approximately 100 nm, and they had the typical bowl-like structure of exosomes. After the exosomes were released using Tris buffer, only a few white particles could be observed on the surface of the chitosan scaffold. These results reveal that the exosome separation device can capture and release exosomes in a controllable manner.

Furthermore, we also used particle size distribution analysis to observe the particle size of the exosomes separated by this device. The results are shown in Figure 2e,f. It can be found that before capture, the particle size of the pretreated medium was particularly inhomogeneous with a large peak around 10 nm and a smaller peak around 100 nm, which indicates that there were a large number of free proteins in the culture medium. After isolation by the device, the size distribution of the exosomes had one large peak at approximately 100 nm and another small peak at approximately 20 nm. This result further verifies that exosomes can be isolated using this device, but there are still a small number of protein impurities in the isolated exosomes. This is mainly because the principle of electrostatic adsorption makes it inevitable that some proteins can also be adsorbed on the surface of chitosan, which may affect the morphology and protein characterization of exosomes. We are now trying to further optimize our equipment such as adding volume exclusion chromatography to pre-remove the protein in the samples.

### 3.3. Characterization of Exosomes Isolated from Serum and Synovial Fluid Using the Exosome Separation Device

**Figure 3:**
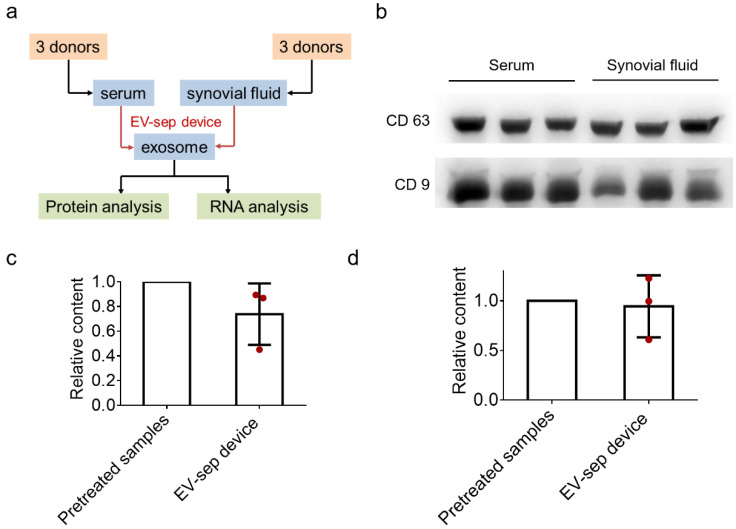
Characterization of protein markers and the RNA content of the obtained exosomes separated by the exosome separation device from serum and synovial fluid. (**a**) A general workflow developed for conducting serum and synovial fluid exosome isolation using the exosome separation device, followed by protein and RNA analysis. (**b**) Expression of CD9 and CD63 in exosomes separated from serum and synovial fluid. (**c**,**d**) The relative content of total RNA in exosomes from serum (**c**) and synovial fluid (**d**) isolated using the exosome separation device (EV-sep device). The results were normalized to the corresponding pretreated samples. The data are presented as the mean ± SD, *n* = 3.

In order to explore the difference and correlation between exosomes of serum and synovial fluid, we used the exosome separation device to isolate exosomes from serum and synovial fluid from six osteoarthritis patients and analyzed their proteins and RNA (Figure 3a). For the protein analysis, we selected CD9 and CD63, two kinds of main protein markers that are usually used to prove the existence of exosomes, to verify the capture of exosome in serum and synovial fluid by Western blot. As shown in Figure 3b, exosomes separated by the device from serum and synovial fluid samples all expressed CD9 and CD63, which indicates that this device can isolate exosomes from serum and synovial fluid successfully. During the experiment, we controlled the initial sample volume of serum and synovial fluid to be 1 mL and ensured that the operation processes of these two body fluids were completely consistent. From the result, there was no significant difference in protein expression between serum and synovial fluid, indicating that there was no significant difference in exosome concentration between serum and synovial fluid.

We also assessed the performance of the device for the extraction of exosomal RNA from serum and synovial fluid. The results are shown in Figure 3c,d. The concentration of RNA obtained by the device was normalized relative to the pretreated samples because of the differences among patients. For serum, the relative content of RNA was 0.74 ± 0.20, and for synovial fluid, the relative content of RNA was 0.94 ± 0.25. These results show that exosomes can be isolated from serum and synovial fluid using this device and RNA can be obtained by in situ lysis.

**Figure 4:**
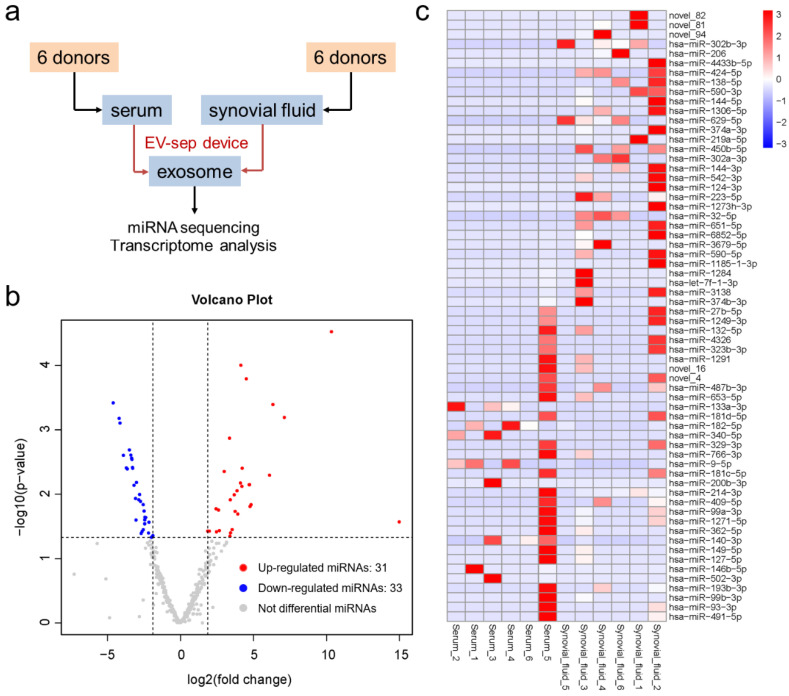
Differentially expressed exosomal miRNAs from the serum and synovial fluid of arthritis patients. (**a**) A general workflow developed for conducting serum and synovial fluid exosome isolation using the exosome separation device, followed by miRNA sequencing and transcriptome analysis. (**b**) Volcano plot of the differentially expressed miRNAs in synovial fluid relative to that in serum. (**c**) Heat map of the differentially expressed miRNAs between the serum and synovial fluid from the arthritis patients.

In order to verify the differences between exosomal miRNAs from serum and synovial fluid, we used this device to isolate exosomes from serum and synovial fluid from 12 osteoarthritis patients, extracting exosomal nucleic acid in situ and sequencing the miRNA (Figure 4a). These 12 patients were all 50–60 years old with no other basic diseases. In this paper, a total number of 921 miRNAs were identified in the serum and synovial fluid exosomes from arthritis patient. The volcanic map shows that there were 31 upregulated and 33 downregulated miRNAs in the synovial fluid compared to the serum of arthritis patients (Figure 4b). The heat map in Figure 4c shows the differentially expressed miRNAs. Among these 64 differentially expressed miRNAs, 59 of them have already been reported, and five of them are identified for the first time in this work (Table 1). It can be seen from the results that there are differences between exosomal miRNAs from serum and synovial fluid. In addition, we are now trying to analyze more samples to discover candidate miRNAs in our next work.

### 3.4. Transcriptome Analysis of Differentially Expressed Exosomal miRNAs in Serum and Synovial Fluid

**Figure 5:**
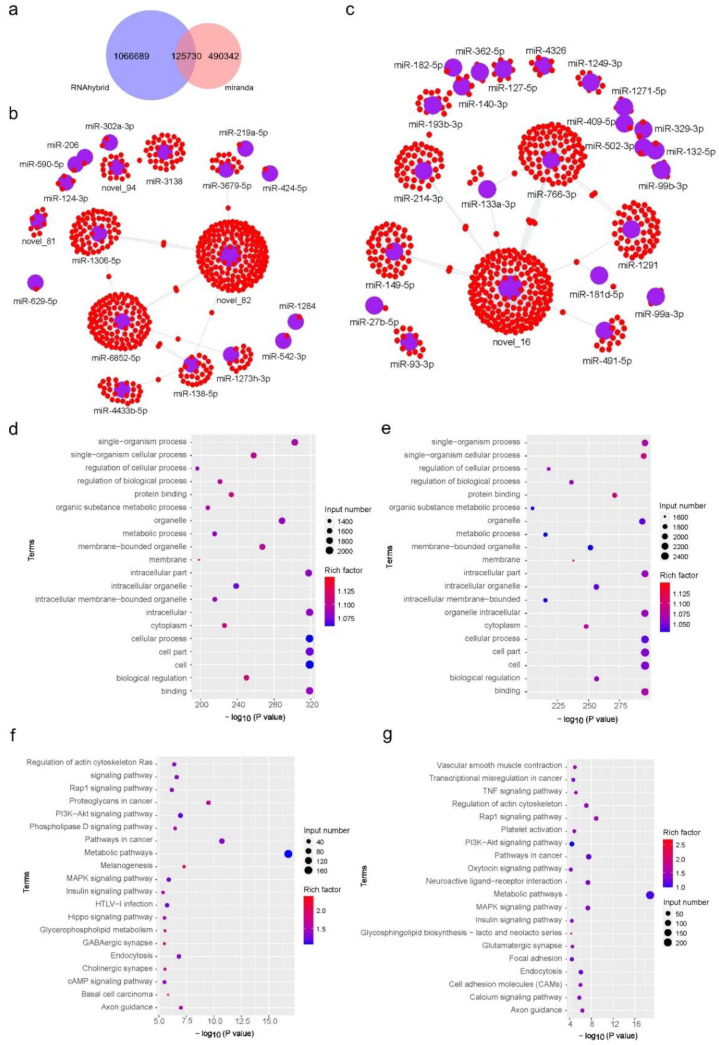
Transcriptome analysis of differentially expressed miRNAs. (**a**) Venn picture of potential target genes by miRanda and RNAhybrid prediction. (**b**) Network of the candidate target genes of upregulated miRNAs. (**c**) Network of the candidate target genes of downregulated miRNAs. The red dots represent candidate target genes, and the blue dots represent miRNAs. (**d**) Enrichment analysis of upregulated expressed genes by GO analysis. (**e**) Enrichment analysis of downregulated expressed genes by GO analysis. (**f**) Enrichment analysis of upregulated expressed genes by KEGG pathway analysis. (**g**) Enrichment analysis of downregulated expressed genes by KEGG pathway analysis.

In order to analyze the differentially expressed miRNAs, the target gene of the 64 exosomal miRNAs was predicted using miRanda as well as RNAhybrid software, and the obtained final potential miRNA target gene set is presented using a Wayne diagram in Figure 5a. Gene network diagrams of upregulated and downregulated genes are shown in Figure 5b,c.

The biological process, molecular function, and cellular component of these up/downregulated potential target genes in synovial fluid were determined by the GO (Gene Ontology) database (Figure 5d,e). Here, we selected the most significant top 20 GO terms by *p*-value for mapping. The results show that the differentially expressed genes of serum and synovial fluid are mainly related to intercellular processes such as cellular processes, binding, and cell parts. In addition, the high rich factor of membrane confirms that there is the expression of genes related to the formation of extracellular vesicles.

We also used KEGG (Kyoto Encyclopedia of genes and genomes) database to determine the main biochemical metabolic pathway and signal transduction pathway of these potential target genes (Figure 5f,g). The results show the top 20 impact pathways. KEGG pathways enrichment reveals that the differential gene are mostly involved in the metabolic pathways, which is caused by the fundamental difference between the two body fluids. Besides, blood related genes such as vascular smooth muscle contraction, platelet activation and so on have been found to be downregulated in synovial fluid. Interestingly, although arthritis is immune associated disease, the result show that cancer related genes such as pathways in cancer, proteoglycans in cancer, basal cell carcinoma and so on have been found upregulated in synovial fluid, which suggests the further mechanism of synovial fluid in immune function remains to be explored.

## 4. Conclusions

In this work, we made the first attempt to compare exosomal miRNAs from serum and synovial fluid in arthritis patients. We observed 31 upregulated and 33 downregulated miRNAs in synovial fluid as compared to serum, which indicated that serum-derived exosomes cannot fully represent the exosomes of synovial fluid. Transcriptome analysis showed that these differentially expressed miRNAs were mainly associated with metabolic pathways and cancer-related pathways. The results may be helpful for the study of joint diseases and the discovery of early diagnostic biomarkers of arthritis.

## Figures and Tables

**Table 1:** The sequence of novel miRNAs.

miRNA	Sequence
Novel_4	CAACGGAAUCCCAAAAGCAGCUG
Novel_16	ACUGCCCCAGGUGCUGCUGGG
Novel_81	UGGGGCGUCGCCAAGUGG
Novel_82	GCAGGCCCGGCGGGGAAGG
Novel_94	GGAGAGGUGGAUGAGUGG

## Data Availability

The data presented in the study are available on request from the corresponding author. The data are not publicly available due to continued analysis by the corresponding author’s research team.
